# Redox regulation of nitrosyl-hemoglobin in human erythrocytes

**DOI:** 10.1016/j.redox.2019.101399

**Published:** 2019-12-05

**Authors:** Flavia Dei Zotti, Roxane Verdoy, Davide Brusa, Irina I. Lobysheva, Jean-Luc Balligand

**Affiliations:** a Institut de Recherche Experimentale et Clinique (IREC), Pole of Pharmacology and Therapeutics (FATH), Cliniques Universitaires Saint-Luc and Université Catholique de Louvain, Brussels, Belgium; b Institut de Recherche Experimentale et Clinique (IREC), Flow Cytometry Platform, Cliniques Universitaires Saint-Luc and Université Catholique de Louvain, Brussels, Belgium

**Keywords:** Nitric oxide, Reactive oxygen species, Erythrocytes, Endothelial dysfunction

## Abstract

Oxidative stress perturbs vascular homeostasis leading to endothelial dysfunction and cardiovascular diseases. Vascular reactive oxygen species (ROS) reduce nitric oxide (NO) bioactivity, a hallmark of cardiovascular and metabolic diseases. We measured steady-state vascular NO levels through the quantification of heme nitrosylated hemoglobin (5-coordinate-α-HbNO) in venous erythrocytes of healthy human subjects using electron paramagnetic resonance (EPR) spectroscopy. To examine how ROS may influence HbNO complex formation and stability, we identified the pro- and anti-oxidant enzymatic sources in human erythrocytes and their relative impact on intracellular redox state and steady-state HbNO levels. We demonstrated that pro-oxidant enzymes such as NADPH oxidases are expressed and produce a significant amount of ROS at the membrane of healthy erythrocytes. In addition, the steady-state levels of HbNO were preserved when NOX (e.g. NOX1 and NOX2) activity was inhibited. We next evaluated the impact of selective antioxidant enzymatic systems on HbNO stability. Peroxiredoxin 2 and catalase, in particular, played an important role in endogenous and exogenous H_2_O_2_ degradation, respectively. Accordingly, inhibitors of peroxiredoxin 2 and catalase significantly decreased erythrocyte HbNO concentration. Conversely, steady-state levels of HbNO were preserved upon supplying erythrocytes with exogenous catalase. These findings support HbNO measurements as indicators of vascular oxidant stress and of NO bioavailability and potentially, as useful biomarkers of early endothelial dysfunction.

## 1 Introduction

Nitric oxide (NO) produced enzymatically by nitric oxide synthase (NOS) in biological systems is a key signaling molecule in the cardiovascular system (reviewed in Refs. [1,2]). Reduced NO production and bioavailability is a major feature of the development of endothelial dysfunction and an accepted hallmark of atherosclerotic cardiovascular diseases and metabolic disorders [3]. Different factors aggravate the development of endothelial dysfunction; in particular, oxidative stress can impair eNOS activity on one hand, and potentiate NO degradation on the other. A proper assay of NO bioavailability in the vasculature could help to better stratify the risk and severity of cardiovascular diseases.

We previously described an assay of the steady-state level of heme nitrosylated hemoglobin in the T (tense) state (5-coordinate-α-HbNO) in venous erythrocytes *in vivo* and proposed it as a surrogate index of vascular NO availability [4]. Circulating NO-donating species can react with deoxygenated hemoglobin within erythrocytes to form this relatively stable complex with heme-Fe(II) which can be quantitatively measured by the Electron Paramagnetic Resonance (EPR) spectroscopy (reviewed in Ref. [5]). In our previous studies, we demonstrated a significant correlation between the HbNO concentrations (quantified in venous erythrocytes) and endothelial function assayed by digital microtonometry in a cohort of healthy volunteers [4], and we proposed that HbNO could be a biomarker to detect endothelial dysfunction at pre-clinical early stage in subjects at risk of developing cardiovascular disease [6]. We showed that despite the expression of a functional NOS in erythrocytes, the NO produced in these cells marginally contributed to the HbNO content compared to NO formed in the vasculature or from exogenous NO donors [7]. However, given the high reactivity of NO, HbNO formation and stability could also be influenced by exogenous or endogenous reactive oxygen species (ROS), and their contribution, as well as the role of the enzymatic antioxidant system in erythrocytes is still unclear.

Indeed, circulating erythrocytes are known to be exposed to intense oxidative stress from both exogenous and endogenous ROS sources that are tightly associated with the primary erythrocyte function to transport and deliver oxygen through the circulatory system to peripheral tissues (reviewed in Ref. [8]). Oxidative stress and its impact on NO bioavailability has been implicated in disorders affecting erythrocytes of patients with sickle cell disease, paroxysmal nocturnal hemoglobinuria, and during the storage of blood for transfusion [9]. ROS produced from white blood cells (neutrophils, monocytes) in plasma, or from endothelial cells in the microvasculature can enter erythrocytes. Additionally, erythrocytes continuously produce intracellular ROS; principally by slow autoxidation of hemoglobin with ensuing formation of methemoglobin (metHb) and superoxide anion (O_2_^.-^), subsequently dismutated to hydrogen peroxide (H_2_O_2_) [10]. Recent studies described the participation of NADPH oxidases (NOXs) in endogenous ROS production in red blood cells (RBCs) from patients with sickle cell disease as well as from healthy subjects, but the impact of their activity on NO metabolism were not clear [11]. On the other hand, erythrocytes carry an extensive antioxidant defense system consisting of non-enzymatic low molecular weight antioxidants such as glutathione and ascorbic acid, and enzymatic antioxidants including superoxide dismutase (SOD), catalase, glutathione peroxidase (GPx1), and different isoforms of peroxiredoxins, e.g. peroxiredoxin-2 (Prdx2) [[12], [13], [14], [15], [16]]. Their role in the preservation of HbNO or NO bioavailability in erythrocytes has never been studied.

This study aimed to characterize the impact of extracellular and intracellular ROS on HbNO formation and steady-state levels in erythrocytes freshly isolated from healthy human volunteers. We determined: a) the expression and activity of selective NOXs isoforms expressed at the erythrocyte membrane or in the cytoplasm, and the interplay of ROS produced by NOXs with HbNO formation; b) the antioxidant enzymes responsible for ROS degradation and protection of HbNO complex formation and steady-state stability; and c) the determinants of the sensitivity of HbNO to extracellular H_2_O_2_. Our results elucidate the mechanisms of the sensitivity of erythrocytes to oxidative stress, as reflected by steady-state levels of HbNO.

## 2 Material and Methods

### 2.1 Blood collection from experimental animals and study subjects

Human blood was collected from healthy volunteers in the morning after a night fasting (n = 15) by a venopuncture from the median cubital vein into vacutainer tubes containing EDTA (K2E, Vacutainer, BD-Plymouth, UK). All volunteers were individually informed about study procedures and signed a written informed consent. The procedures were approved by the local Ethics Committee of the Faculty of Medicine and Saint Luc university hospital of the Université Catholique de Louvain.

### 2.2 Isolation of red blood cells and ghost membrane preparation

Human erythrocytes were collected after blood centrifugation at 800–1000×*g* (10 min at 4 °C) and three consecutive washing with an isotonic buffer (phosphate buffered saline, PBS, with 5 mmol/L glucose, pH 7.4) followed by centrifugation at 800×*g* (5 min), then processed for EPR measurements of HbNO, for immunofluorescence and flow cytometry analysis. The residual content of white blood cells (WBCs) in RBCs fraction was tested by flow cytometry. Whole blood and isolated RBCs samples were incubated with goat serum for 15 min and then with anti-CD235a (detecting erythrocyte glycophorin; 130-117-800 Miltenyi Biotec) and anti-CD45 (detecting leukocyte common antigen; 130-110-773 Miltenyi Biotec) antibodies for 30 min at room temperature. Samples were washed in PBS at 200×*g* for 5 min and ultimately suspended in PBS for data acquisition performed with FACSCantoII™ flow cytometer (BD Biosciences) and data analysis with FlowJo software (BD Biosciences).

To isolate ghost membranes, human RBCs were lysed with a cold hypotonic buffer (0.5 mmol/L Tris-HCL, 0.05 mmol/L dithiothreitol DTT, and 1 mmol/L phenylmethylsulfonyl fluoride, pH 8.5) with protease inhibitor cocktail (P8340, Sigma) at 1:20 (v:v) ratio, and incubated at 4 °C for 30 min and at 37 °C for 15 min. Cytosolic fractions were collected by ultracentrifugation (30,000×*g* for 35 min at 4 °C) from the supernatant and the pellet was divided in two tubes after re-suspension in 10 mmol/L Tris-HCl and 0.5 mmol/L EDTA, pH 8, ultracentrifuged, and washed twice in 10 mmol/L Tris-HCl (pH 7.4); one tube with white ghost membranes was used for ROS detection with EPR spin probe (1-Hydroxy-3-carboxy-2,2,5,5-tetramethylpyrrolidine-HCl, CPH hydrochloride, purchased from EnzoLife Sciences, ALX-430-078), and the pellet from the second tube was suspended into 20 mmol/L Tris-HCL, 0.1 mmol/L DTT and 250 mmol/L d-glucose, pH 7.4, ultracentrifuged and collected in a lysis buffer (20 mmol/L EGTA, 15 mmol/L MgCl_2_, 80 mmol/L β-glycerophosphate, 10% of Triton-X100, 0.5% of Glycerol and with protease inhibitor cocktail) for Western blot analysis. Human RBCs, white blood cells (WBCs), human umbilical vein endothelial cells (HUVEC) and human embryonic kidney cells (HEK293) were lysed and collected in RIPA buffer (50 mmol/L Tris, 150 mmol/L NaCl, 1% Triton-X-100, 0.05% sodium deoxycholate, 1 mmol/L EDTA, 0.1% SDS, pH 7.4) with protease inhibitor cocktail. Lysate, cytosolic and membrane fractions were sonicated and centrifuged at 10,000×*g* for 10 min to remove cell debris. Total protein concentrations were determined by the Bradford assay (5000006, BioRad).

### 2.3 Hemolysis assay

We verified the effect of drug treatments on hemolysis rate of human RBCs as previously described [17]. Isolated erythrocytes were suspended in Krebs-Henseleit buffer at 1:50 (v:v) ratio (in mmol/L: 100 NaCl; 5 KCl; 1 KH_2_PO_4_; 1.2 MgSO_4_; 25 NaHCO_3_; 5 d-glucose; 20 Hepes; and 0.01 diethyldithiocarbamic acid, sodium salt, DETC, pH 7.4). Erythrocytes were incubated at 37 °C for 1 h in a V-bottom 96-well plate, using an orbital shaker, with different drugs: 10 μmol/L of 3-AT (3-Amino-1,2,4-triazole; A8056 Sigma); 25 μmol/L of ConoidinA (18080-67-6, Cayman Chemical); 10 μmol/L of H_2_O_2_; 110 nmol/L GKT137831 (S7171 Selleckchem); 770 nmol/L VAS2870 (Sigma-Aldrich); 50 units/mL of Catalase (C9322, Sigma); 50 units/mL of SOD (S7571 Sigma-Aldrich). As positive control, we incubated RBCs with 20% of Triton X-100. The plate was then centrifuged at 500×*g* for 5 min to pellet intact RBCs. Supernatant samples were transferred into a clear plate and the absorbance was measured at 541 nm with a Spectramax i3 (Molecular Devices, LLS, USA).

### 2.4 Superoxide anion measurements by EPR spectroscopy

15 μg of erythrocyte white ghost membranes collected into 10 mmol/L Tris-HCl (pH 7.4), were incubated for 10 min with SOD (30 units/mL) or with inhibitors of specific isoforms of NOX: 110 nmol/L, or 140 nmol/L, or 410 nmol/L of GKT137831 to inhibit NOX1, NOX4, NOX5, respectively, and VAS2870 770 nmol/L to inhibit NOX2; l-NAME (Nω-Nitro-l-arginine methyl ester hydrochloride, 5 mmol/L, N5751, Sigma) was used to inhibit NOS. NOXs cofactor, β-NADPH (100 μmol/L, N7505 Sigma-Aldrich), and the EPR spin probe, CPH (6.7 mmol/L) were added just before the EPR measurements. The CPH was dissolved in Krebs-Henseleit-DTPA buffer (in mmol/L: 100 NaCl; 5 KCl; 1 KH_2_PO_4_; 1.2 MgSO_4_; 25 NaHCO_3_; 5 d-glucose; 20 Hepes; 0.1 diethylenetriaminepentaacetic acid, DTPA; and 0.01 diethyldithiocarbamic acid silver salt, DETC, pH 7.4) that was pre-incubated for 1 night at 1% of O_2_ in hypoxia workstation in order to avoid spin probe oxidation. The EPR signals of CP^**.**^ radicals formed in the samples after reaction of CPH with superoxide anions were recorded using an EPR spectrometer (X-band, Magnettech, MS400) at 20 °C; the linear slope was calculated from the kinetics of CP^**.**^ radicals formation from 5 repeated measurements during 10 min after addition of CPH for each sample; and was used for quantitative assay of superoxide anion production after subtraction of the basal CP^**.**^ signal. The parameters of the EPR spectrometer for the spectra recording were: microwave frequency ~9.35 GHz; modulation frequency (MF), 100 kHz; microwave power (MW), 10 mW; modulation amplitude (MA), 2 G.

### 2.5 Western blot analysis

For detection of the NADPH oxidase isoforms, proteins (50 μg) from human RBC lysate, cytosol and ghost membranes were first denatured and then loaded and separated on 10% SDS polyacrylamide gel (Bio-Rad) and transferred to nitrocellulose membranes. Alternatively, to determine the antioxidant enzymes 20 μg of proteins were subjected to electrophoresis on 12.5% SDS polyacrylamide gel (Bio-Rad) and transferred to PVDF membranes. Membranes were blocked with 1% nonfat dry milk in Tris-buffer saline solution with 0.1% Tween 20 (TBST, Sigma), incubated overnight with primary antibody at 4 °C, washed in TBST and then incubated for 1 h with goat anti-mouse or goat anti-rabbit antibody (1:5000, Jackson). Immunoblotted bands were detected by an Amersham Imager 600 (GE Healthcare Life Sciences) using chemiluminescent reagents: HRP substrate (Millipore) [11] or CDP-Star substrate for alkaline phosphatase (WB7106, Invitrogen). The following primary antibodies were utilized: anit-NOX1 (ab55831, Abcam), anti-NOX2/gp91phox (ab80508, Abcam), anti-NOX4 (ab133303, Abcam), anti-NOX5 (ab198213, Abcam), anti-SOD1 (sc-8637, Santa Cruz), anti-Catalase (ab16731, Abcam), anti- GPX1 (ab22604, Abcam), anti-Prdx6 (ab16824, Abcam), anti-AQP1 (AB2219, Millipore) and anti-Prdx1 and Prdx2 were gently provided by Prof. B. Knoops (Louvain Institute of Biomolecular Science and Technology, UCLouvain, Belgium).

### 2.6 Immunofluorescence microscopy and flow cytometry

Freshly isolated erythrocytes (directly after plasma separation) were smeared across glass slides and fixed with cold 4% paraformaldehyde for 30 min on ice. Erythrocytes were immunoblotted, after extensive washing, with an anti-SOD3 (sc-67089, Santa Cruz) and anti-CD235a (555569, BD) antibodies for all night at 4 °C, and subsequently washed and incubated with secondary antibodies Alexa-488 goat anti-rabbit and Alexa-568 anti-mouse (Invitrogen) for 1 h. The preparations were mounted in fluorescent counting medium (DAKO) and examined in a fluorescence microscope equipped with an ApoTome module for structured illumination (Zeiss AxioImager.z1, USA).

For flow cytometry analysis 40 μl of RBCs (5 × 10^8^ cells), collected directly after plasma separation, were fixed overnight in 1 mL of paraformaldehyde 4% at 4 °C followed by washing with staining buffer (PBS with 1% FBS, 0.09% NaN_3_) and incubation with anti-SOD3 and with anti-CD235a antibodies for 30 min at room temperature. Samples were washed in PBS (with centrifugation at 200×*g*, 5 min) and incubated with anti-mouse or anti-rabbit Alexa Fluor-conjugated secondary antibodies for 20 min at RT. RBCs were suspended in staining buffer, data acquisition was performed with a FACSCantoII™ flow cytometer (BD Biosciences) and data analysis with FlowJo software (BD Biosciences).

### 2.7 Measurements of erythrocyte HbNO

To measure the steady-state levels of HbNO in human erythrocytes after incubation with a NO-donor, all the samples were frozen in calibrated tubes in liquid nitrogen for low-temperature EPR measurements. EPR spectra were recorded using the X-band EPR spectrometer (MS400, Magnettech) at 77 K using an EPR quartz finger Dewar filled with liquid nitrogen with the following settings: microwave frequency ~ 9.35 GHz; MF, 100 kHz; MW, 20 mW; MA, 7 G, 5 scans. The quantification of HbNO concentrations was performed as described before [7]. Briefly, the HbNO EPR spectrum displays the well resolved triplet hyperfine structure (hfs) A_hf_ = 16.8 G, at g_z_ ~ 2.01 and the peak-to-peak amplitude of the hfs component was used for 5-coordinate α-HbNO quantification. The calibration curve for HbNO quantification was obtained as described previously [7] using the HbNO complexes synthesized at different concentrations after incubation of erythrocytes with NO-donor system (gradually diluted solution of sodium nitrite and 50 mmol/L of sodium dithionate) in anaerobic condition. The real concentration of synthesized HbNO in these samples was quantified by comparison of the signal intensity obtained by spectra double integration with that of a common EPR standard (Cu[EDTA]_5_ complex, 50 and 100 μmol/L frozen in 30% glycerol-water solution).

### 2.8 Measurement of the influence of NADPH oxidases or antioxidant enzymes on HbNO stability by EPR spectroscopy

Human blood daily collected was centrifuged and incubated at 37 °C in hypoxia workstation (INVIVO_2_400, Ruskinn Technology, Ltd, UK) at 4% of O_2_ where plasma was removed. The erythrocytes were reconstituted at 50% of haematocrit in Krebs-Henseleit buffer, incubated and continuously mixed with 50 μmol/L of NO-donor((Z)-1-[N-[3-aminopropyl]-N-[4-(3-aminopropylammonio)butyl]-amino]diazen-1-ium-1,2-diolate, Spermine-NONOate, Enzo Life Science) for 45 min. A stock solution of the NO-donor was prepared from Spermine-NONOate powder dissolved in NaOH (0.01 M), and diluted in physiological solution (pH 7.4) before immediate addition to cells in hypoxia chamber. The final concentration of 50  μM was chosen based on the concentration-response curve as illustrated in Fig. 4D for optimal signal/noise ratio. Treatment with inhibitors of NOX 1, NOX 2, NOX 4, or vehicle was provided for 30 min at 4% of O_2_, and the RBCs were frozen in calibrated tubes in liquid nitrogen every 15 min during next 45 min for low-temperature EPR measurements.

The antioxidant enzymes (catalase and Prdx2) were inhibited respectively by 10 μmol/L of 3-AT and by 25 μmol/L of ConoidinA for 30 min at 4% of O_2_ in RBCs (under continuous mixing) after reconstitution (50% of haematocrit) and Spermine-NONOate addition as described above. Then 10 μmol/L of H_2_O_2_ was added for 15 min incubation at 4% of O_2_ and the samples were frozen every 15 min (up to 45 min) for the EPR measurements.

### 2.9 Measurement of exogenous catalase and SOD on HbNO stability by EPR spectroscopy

To study the accumulation of HbNO upon incubation with a NO donor, RBCs were collected from venous blood, washed and reconstituted at 50% of haematocrit with isotonic buffer. Erythrocytes were incubated with 50 units of non-PEGylated catalase (C9322, Sigma) or non-PEGylated SOD (S7571, Sigma) at 37 °C during 1 h in a hypoxia workstation (RUSKINN INVIVO_2_300) at 1% of O_2_ and at room air (21% of O_2_). Then, different concentrations of Spermine-NONOate (10-20-50 and 100 μmol/L) were added for 45 min, samples were incubated at 1% of O_2_ in hypoxia chamber under continuous mixing (with Eppendorf Thermomixer) and afterwards frozen in calibrated tubes in liquid nitrogen for low-temperature EPR measurements.

To study the influence of catalase on HbNO stability, reconstituted RBCs were firstly incubated with 50 μmol/L of NO-donor for 15 min and then with 50 units/ml of catalase for 30 min in hypoxia workstation at 4% of O_2_. Samples were subsequently treated with H_2_O_2_ for 15 min and were collected every 15 min until 45 min.

### 2.10 ROS detection by flow cytometry in RBCs

Isolated human erythrocytes were re-suspended (5 × 10^6^ cells) in Hank's Buffered Saline Solution (HBSS, Lonza Bioscience) and incubated for 30 min at room temperature and at 4% of O_2_ with the following reagents: 3-AT (10 μmol/L), ConoidinA (25 μmol/L), catalase (50 units/ml). CM-H_2_DCFDA (DCFDA; C6827, ThermoFisher Scientific) at 1 μmol/L was added to each sample for additional 25 min incubation at 37 °C and 4% of O_2_. Samples were washed twice with HBSS and at least 10000 events were analyzed for fluorescence on FACSCantoII™ flow cytometer at room oxygen. For the experiments with antioxidant inhibitors, 10 μmol of H_2_O_2_ was added after a first reading and a kinetic curve of ROS formation was measured every 5 min until 45 min for each sample.

### 2.11 Statistics

Results are shown as mean values ± SEM. Data were statistically analyzed as indicated in the figure legends, using GraphPad Prism 7.01, after verification of normality of the value distributions (using the Kolmogorov–Smirnov test). The SAS 9.4 software was used for mixed model statistical analysis. Median fluorescence intensity (MFI) values were obtained from at least 10000 events analyzed for fluorescence on FACSCantoII™ flow cytometer and normalized in each experiment to a baseline of control unstained samples. Differences were considered statistically significant with a P-value < 0.05.

## 3 Results

### 3.1 Erythrocyte enzymatic sources of ROS and their effect on HbNO stability

#### 3.1.1 NADPH oxidase expression and production of ROS in human isolated RBCs

**Fig. 1:**
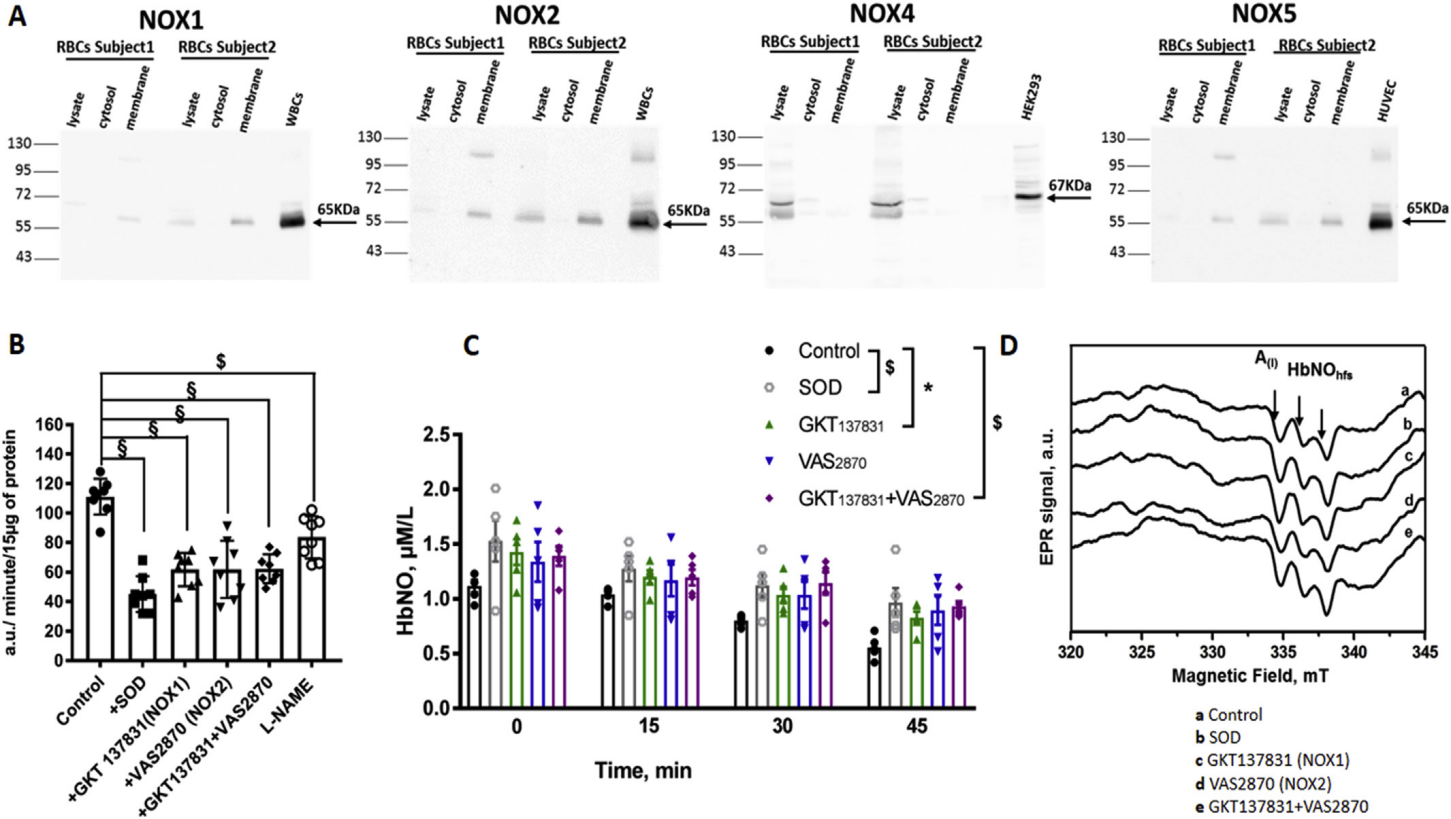
Erythrocyte enzymatic sources of ROS and their contribution to HbNO stability. A) Representative western blots from total RBCs lysates, RBC cytosolic fractions, and isolated ghost membranes (50 μg of protein each) reveal the expression of NOX isoforms 1, 2, 4 and 5 in human RBCs from healthy subjects. NOX1, NOX2 and NOX5 isoforms were observed at ~65 kDa in membrane fractions; NOX4 isoform was observed in cytosolic fraction at ~67 kDa. Human WBCs lysate was loaded as a positive control for NOX1 and 2; HEK293 cell lysate for NOX4; and HUVEC cell lysate for NOX5. Chemiluminescent HRP substrate was used for immunoblotted band detection. B) Effects of SOD (50 units/mL), and NOX inhibitors (GKT137831, 110 nmol/L; VAS2870, 770 nmol/L), or both (GKT137831 + VAS2870) and l-NAME (5 mmol/L) on O_2_^.-^ formation quantified by the EPR spin probe (CPH) in isolated ghost membranes. The linear slope of increase of the CP^.^ EPR signal during 10 min after addition of the spin probe to the sample was measured and reported as rate of O_2_^.-^ production normalized by protein content. C) HbNO concentration in human RBCs (50% haematocrit) obtained after incubation with Spermine-NONOate (50 μmol/L) during 45 min under 4% O_2_ level in the absence (Control) or co-incubation with SOD, or NOX inhibitors, as in B. After 45 min of pre-incubation, samples were collected every 15 min until 45 subsequent minutes (at 4% O_2_), and were frozen in liquid nitrogen for the low-temperature EPR assay. D) Typical EPR spectra obtained from human RBCs incubated with the NO-donor (50 μmol/L; during 45 min at 4% of O_2_) and frozen after subsequent 30 min treatment (at 4% of O_2_) with a) control (vehicle); b) SOD; c) GKT137831; d) VAS2870; or e) with both GKT137831 and VAS2870, as in B. The hyperfine structure (hfs) of the HbNO EPR signal is shown by the arrows; A(I) indicates the amplitude of the first hf component. The EPR spectra were recorded using the EPR spectrometer (MS400, Magnettech) as described in Material and Methods. Data in B and C are shown as mean values ± SEM and were treated using a mixed model statistical analysis with Dunnett's adjustment for multiple comparisons; *P < 0.05 ^$^P < 0.01 ^§^P < 0.001; n = 8 different preparations of RBC ghost membrane (B) and n = 5 different RBC preparations (C).

We first investigated potential enzymatic sources of ROS production in human RBCs from healthy subjects by performing Western blot analysis of the NADPH oxidase isoforms distributed in different erythrocyte compartments. Cell lysates, cytoplasm and “white ghost” membranes from erythrocyte fractions were analyzed for the subcellular localization of the different NOX isoforms; NOX1, NOX2 and NOX5 were detectable in cell lysates and in membrane fractions, while NOX4 was in whole lysate and cytosolic fraction only (Fig. 1A). We confirmed the NOX2 signal observed in membrane fraction by HRP-based detection using the alkaline phosphatase as alternative chemiluminescent substrate (Supp. figure 1A). We next measured the O_2_^.-^ formation using the EPR spin probe, CPH in ghost membranes isolated from healthy human RBCs. Erythrocyte membrane samples were pretreated with vehicle or SOD or NOX inhibitors, or with l-NAME, an inhibitor of nitric oxide synthase (NOS) (Fig. 1B). SOD, an antioxidant enzyme that catalyzes the dismutation of superoxide radicals to H_2_O_2_ and molecular oxygen, reduced detection of O_2_^.-^ in membrane samples by 60% from control values. Since eNOS is expressed at the membrane of human erythrocytes [7,18], we tested the effect of l-NAME which can inhibit not only NO production but also the O_2_^.-^ production formed from uncoupled eNOS. We found that l-NAME decreased the O_2_^.-^ accumulation by 25% compared to control samples (Fig. 1B). We tested further the effect of NOX inhibitors. GKT137831 is a dual inhibitor of both NOX1 and NOX4 with K_i_ values of 110 and 140 nmol/L but it was also described to inhibit NOX5 (at higher K_i_ values of 410 nmol/L). VAS2870 preferentially inhibits NOX2 with K_i_ value of 770 nmol/L. Inhibition of NOX1 and/or NOX2 produced a significant reduction in membrane O_2_^.-^ production by about 44%; these effects were comparable to that of SOD (Fig. 1B).

#### 3.1.2 HbNO stability is improved by inhibiting NADPH oxidases

The erythrocyte membrane NOX1 and/or NOX2 generated a significant portion of ROS. Therefore, we tested the stability of HbNO after inhibition of these NOXs. Note that erythrocytes were incubated at 4% of O_2_ (venous oxygen level) where hemoglobin is mostly in T-state conformation amenable to HbNO complex formation. Isolated erythrocytes were pre-incubated with a NO donor and pre-treated with the NOX inhibitors, VAS2870 or GKT137831, or both, or with SOD before freezing samples for low temperature EPR measurements. The calculated HbNO concentrations and typical EPR spectra are shown in Fig. 1C and D. Combining both inhibitors increased the stability of HbNO compared to control samples (66.6 ± 0.1% augmentation after 45 min), grossly to the same extent as the addition of exogenous SOD (73.4 ± 0.3% augmentation after 45 min. We excluded any potential leukocyte contamination in isolated erythrocyte preparations by flow cytometry analysis of the samples after staining with CD235a and CD45 antibodies, specific for erythrocytes and leukocytes respectively (Supp. figure 1B; whole blood was used as positive control). Moreover, no hemolysis was observed after the addition of NOX inhibitors, SOD or catalase to erythrocytes (Supp. figure 1C).

### 3.2 Endogenous antioxidant enzymatic systems influence the steady-state levels of HbNO in human erythrocytes

#### 3.2.1 Characterization of the enzymatic antioxidant system of human erythrocytes

**Fig. 2:**
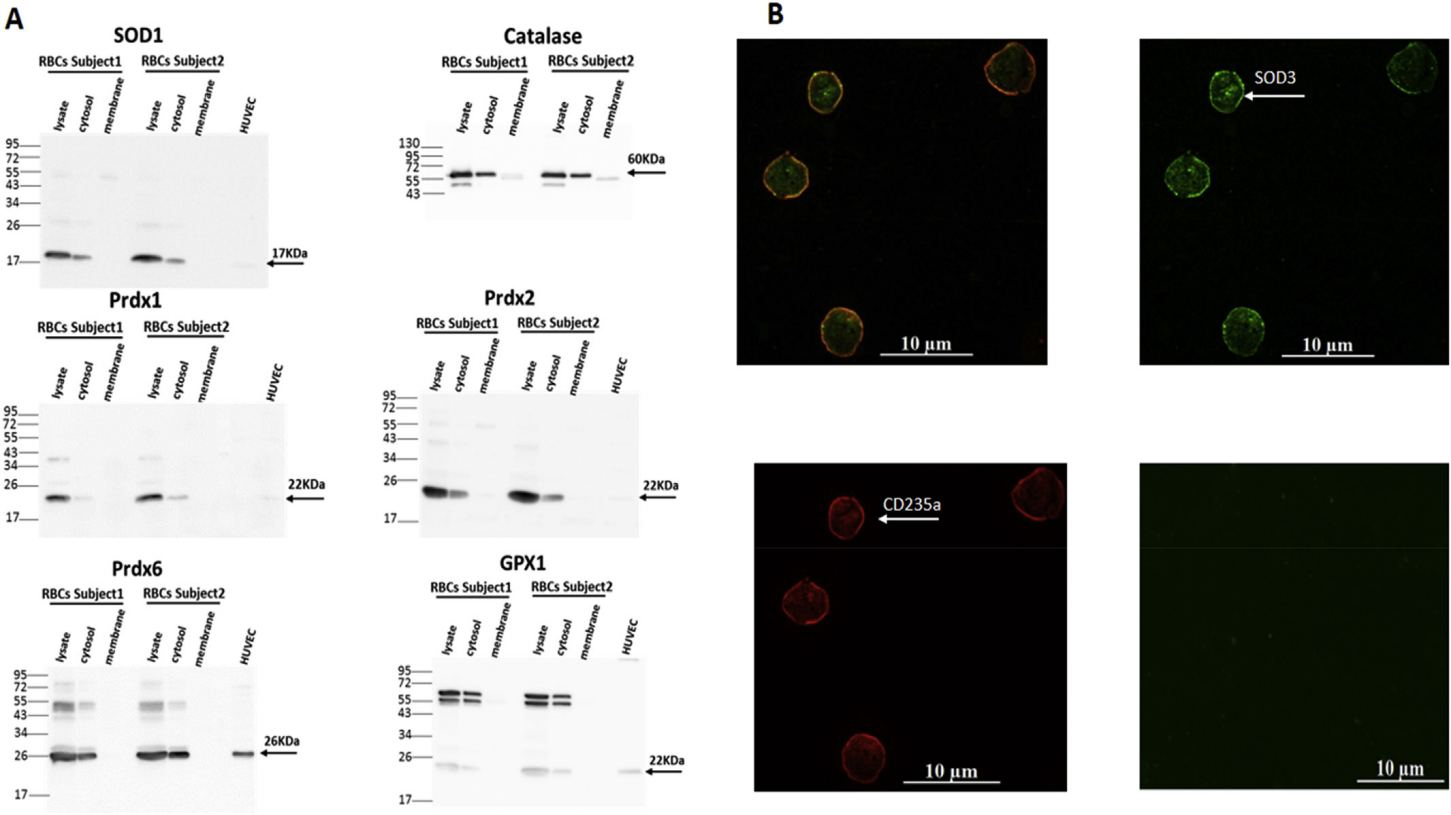
Antioxidant enzymatic systems in human RBCs. A) Representative Western blot analysis of antioxidant enzymes expressed in the cytosol of human RBCs. Human total erythrocyte lysates, cytosolic fractions and isolated ghost membrane samples (20 μg of proteins each) were analyzed by immunoblotting analysis as described in Materials and Methods; bands specific for SOD1 (at 17 kDa); Catalase (at 60 kDa); GPX1, Prdx1 and 2 (at 22 kDa); and Prdx6 (at 26 kDa) are shown using chemiluminescent HRP-based detection. HUVEC cell lysates were used as positive control (except catalase). B) Representative image of immunofluorescent detection of SOD3 (upper-right panel) and CD235a (or Glycophorin A; lower-left panel) in healthy human RBCs as described in Materials and Methods. The upper-left panel shows the overlay of both immunodetected signals. As negative control (bottom-right panel), RBCs were stained with conjugated secondary antibody only (Alexa Fluor-488 anti-mouse IgG).

RBCs have an extensive antioxidant system as demonstrated by our Western blot analysis illustrated on Fig. 2. We detected immunoblotted signals for SOD1 in whole RBC lysates and cytosolic fractions (Fig. 2A); instead SOD3, known as the extracellular isoform, can be attached to the erythrocyte membranes, as illustrated by its immunofluorescence detection at the surface of human RBCs (Fig. 2B), and by flow cytometry (Supp. figure 2A). In these experiments, the erythrocytes were collected after plasma separation without extensive washings in order to preserve all the membrane-associated proteins, and they were double-stained for SOD3 and CD235, or glycophorin A, selectively identifying erythrocytes. Flow cytometry revealed only a limited amount of erythrocytes to be positive for SOD3 (as shown by a shift from the bottom-left quadrant, when they were stained only with secondary antibodies, to the upper-right quadrant of the panel in Supp. figure 2A). In addition, immunoblotted signals for Catalase, Peroxiredoxin 1, 2 and 6 and Glutathione peroxidase 1 were found predominantly in cytosolic and whole lysate fractions of erythrocytes, as shown by western blotting in Fig. 2A (HRP-based detection), and confirmed with immunoblotted band detection by alkaline phosphatase (Supp. figure 1A).

#### 3.2.2 The erythrocyte antioxidant system preserves HbNO stability by neutralizing ROS produced intra- and extracellularly

**Fig. 3:**
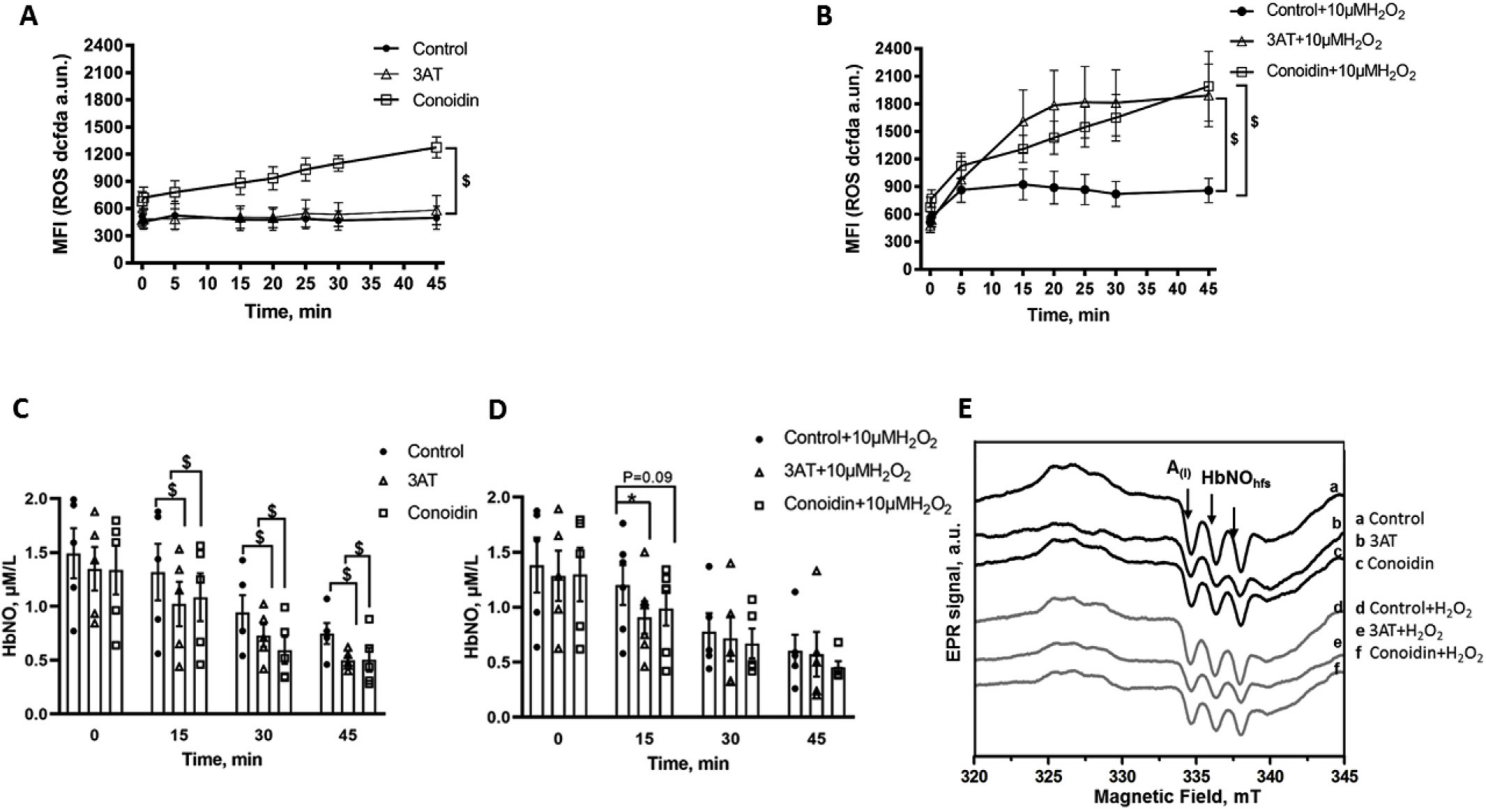
Role of antioxidant enzymatic systems in human RBCs. A-B) Effect of inhibition of catalase (with 3-AT; 10 μmol/L) or Prdx2 (with ConoidinA 25 μmol/L) on ROS production in human RBCs. Mean fluorescence intensity (DCFDA 1 μmol/L) was measured every 5 min after addition of (A) vehicle or (B) H_2_O_2_ (10 μmol/L) during 45 min in erythrocytes pre-incubated with inhibitors for 10 min. C-D) HbNO concentration in human RBCs (at 50% haematocrit) pre-incubated with Spermine-NONOate (50 μmol/L) in the absence (Control) or presence of inhibitors of catalase or Prdx2 (for 45 min under 4% of O_2_); RBCs were subsequently exposed to vehicle (C) or H_2_O_2_ (D) for 15–45 min (at 4% of O_2_) and frozen for low-temperature EPR assay as described in Materials and Methods. E) Typical EPR spectra recorded from human RBCs pre-treated with Spermine-NONOate (50 μmol/L) with/without 3AT or ConoidinA, then exposed to H_2_O_2_ (d-e-f) or vehicle (a-b-c) for 15 min and frozen for EPR assay. The hyperfine structure (hfs) of the HbNO EPR signal is shown by the arrows. Data in A, B, C and D are shown as mean values ± SEM and treated using a mixed model statistical analysis with Dunnett's adjustment for multiple comparisons; *P < 0.05 ^$^P < 0.01; respectively n = 5 different RBC preparations.

We have shown that SOD decreased the production of O_2_^.-^ in erythrocyte membranes and preserved HbNO; however SOD activity was expected to proportionally increase the content of H_2_O_2_, another ROS source, as product of its dismutase enzymatic activity. We then investigated the potential role of the other intracellular antioxidant enzymes, as identified above, on ROS and HbNO contents in erythrocytes. In particular, we inhibited peroxideroxin2, the most abundant antioxidant enzyme expressed in these cells [19] with Conoidin A, and catalase with 3-AT. ConoidinA inactivates peroxiredoxin2 by covalently binding to the catalytic cysteine on the enzyme (IC_50_ = 23 μmol/L) [20,21], while 3-AT binds covalently the active center of the catalase tetrameric form [22]. Fig. 3A and B illustrate the effect of Prxd2 or catalase inhibition on ROS formation (DCFDA) in RBCs in absence (3A) or in presence (3B) of an oxidative challenge during exposure to H_2_O_2_. In absence of added H_2_O_2_, inhibition of Prdx2, but not catalase, produced an increase of intracellular ROS production in RBCs (96% increase at 20 min) compared with control cells. However, in RBCs exposed to exogenous H_2_O_2_, both inhibition of catalase and Prdx2 produced a strong increase of intracellular DCFDA signal, with catalase inhibition producing a doubling of the signal at 20 min (Fig. 3B), which was maintained at 45 min Prdx2 inhibition produced a 60% increase of ROS at 20 min of H_2_O_2_ addition which increased up to 132% compared to control at 45 min (Fig. 3B). We next determined the contribution of Prdx2 and catalase on HbNO stability in similar healthy human RBCs at 4% of O_2_ (equivalent to venous pO_2_ level). The HbNO EPR signal decreased with time significantly more in erythrocytes treated with 3-AT or ConoidinA by comparison with untreated RBCs (Fig. 3C). This difference seemed to be abrogated under treatment with exogenous H_2_O_2_, which uniformly produced a decay of HbNO over time, except that it was significantly more pronounced under catalase inhibition at 15 min (Fig. 3D; by 31.2 ± 0.4% for 3-AT, and 18.3 ± 0.4% for ConoidinA compared with control). Fig. 3E displays the typical EPR spectra of HbNO recorded at 15 min with/without H_2_O_2_ addition in RBCs treated with vehicle (a,d), 3-AT (b,e) or ConoidinA (c,f).

**Fig. 4:**
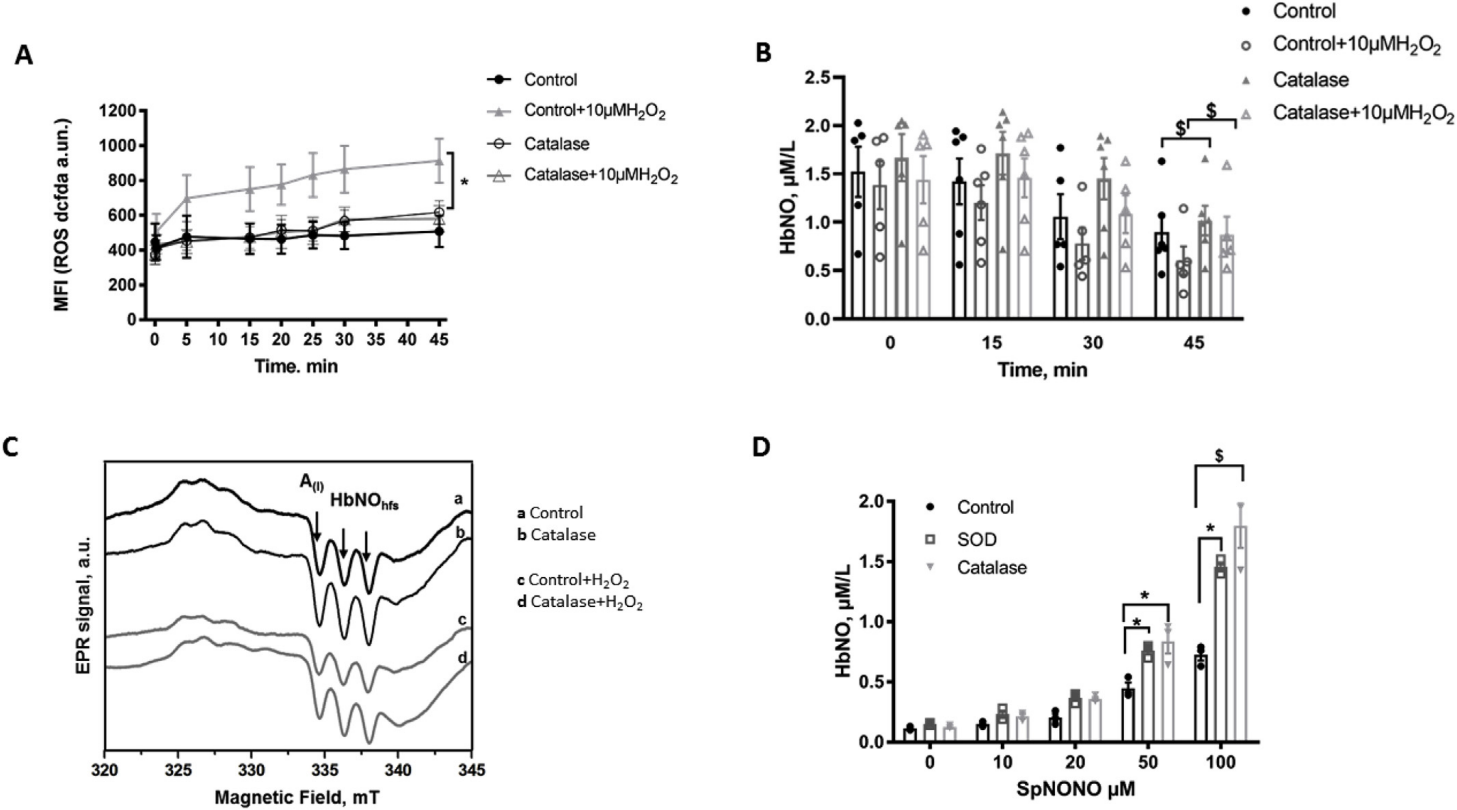
Effect of extracellular catalase on ROS and HbNO formation in human RBCs. A-B) Effect of catalase on ROS formation (A) in total erythrocytes assayed by DCFDA (1 μmol/L) fluorescence; and on HbNO concentrations (B) assayed by EPR in human RBCs (50% haematocrit) pre-incubated with Spermine-NONOate (50 μmol/L; 45 min). Non-PEGylated catalase was pre-incubated under venous O_2_ level (4% O_2_; 45 min), then H_2_O_2_ (10 μmol/L; or vehicle) added and fluorescence measured every 5 min. (A); or erythrocytes were frozen every 15 min up to 45 min for low-temperature EPR spectroscopy (B). C) Typical EPR spectra recorded in human RBC samples frozen at 30 min after pre-treatment with 50 μmol/L of Spermine-NONOate, without (a–c) or with (b–d) catalase (45 min at 4% of O_2_), and exposed to vehicle (a–b) or H_2_O_2_ (c–d). The hyperfine structure (hfs) of the HbNO EPR signal is shown by the arrows. D) Erythrocyte HbNO concentrations quantified from EPR signals of RBCs (50% of haematocrit in isotonic buffer) pre-incubated with antioxidant enzymes SOD or catalase at 21% of O_2_ and then, exposed to graded concentrations of Spermine-NONOate for 45 min at 1% of O_2_ before freezing for the low-temperature EPR assay. Data in A, B and D are shown as mean values ± SEM and treated using a mixed model statistical analysis with Dunnett's adjustment for multiple comparisons; *P < 0.05 ^$^P < 0.01; n = 3–6 different RBC preparations.

These data pointed to catalase as a critical antioxidant enzyme to preserve the intracellular milieu and HbNO content from an oxidant challenge with exogenous H_2_O_2_. To confirm this, we tested the effect of exogenous, non-PEGylated catalase on ROS accumulation (Fig. 4A), HbNO stability (Fig. 4B and C) and HbNO formation under extracellular NO administration (Fig. 4D). Extracellular (i.e. cell-impermeable) catalase had no influence on intracellular ROS (DCFDA) in absence of added H_2_O_2_ (Fig. 4A). In presence of an oxidant challenge (10 μmol/L of extracellular H_2_O_2_) catalase significantly decreased ROS accumulation intracellularly (by 37 ± 0.3% at 20 min compared to control samples) (Fig. 4A). Consistent with its effect to decrease ROS, catalase increased the stability of HbNO over time (e.g. at 45 min, Fig. 4B), as also illustrated in representative EPR spectra in Fig. 4C. In addition, we directly compared the effect of extracellular catalase with that of extracellular (non-PEGylated) SOD on the formation of HbNO in human erythrocytes incubated with graded NO-donor concentrations for 45 min. We observed that while both enzymes had a prominent effect to potentiate HbNO formation under oxygenated condition, the steady-state concentration of HbNO was higher in presence of catalase at 50 and 100 μmol/L of NO-donor (88.5 ± 0.2% increase by catalase over control vs. 70 ± 0.6% by SOD at 50 μmol/L of NO donor; and 2.47 times control values with catalase vs. 2.0 with SOD at 100 μmol/L of NO donor). Conversely, erythrocytes incubated at 1% of O_2_ showed no difference in HbNO accumulation after treatment with extracellular SOD or catalase (Supp. figure 2B) suggesting that extracellular ROS species are produced by RBCs in oxygenated conditions, and neutralized by SOD and by catalase extracellularly. Of note, all the added drugs (ConoidinA, 3-AT, SOD and catalase) did not increase hemolysis under similar conditions (Supp. figure 1C).

## 4 Discussion

The main findings from our study can be summarized as follows; i. NOX1, NOX2 and uncoupled eNOS, are the main sources of O_2_^.-^ in membrane extracts of human erythrocytes; ii. O_2_^.-^ produced by NOX1-2 decreases steady-state levels of HbNO; iii. among the anti-oxidant armamentarium of RBCs, cytosolic peroxiredoxins (e.g. Prxd2) attenuate the constitutive intracellular production of oxidant species, while both Prdx and catalase cooperate to reduce the intracellular effect of exogenously applied H_2_O_2_; iv. the effect of extracellular (exogenously added), but also endogenous catalase (also expressed at the membrane) against degradation of HbNO points to extracellularly generated H_2_O_2_ as a significant oxidant species influencing steady-state HbNO levels.

We previously demonstrated that the paramagnetic complex of 5-α-nitrosyl-hemoglobin mainly originates from the exposure of erythrocytes to paracrine sources of NO-donating species and that its abundance in venous erythrocytes correlates with endothelium-dependent vasodilatation [4,7]. In parallel, it also negatively correlated with circulating plasma peroxides, suggesting that it may be a surrogate index of the oxidant stress that generates endothelial dysfunction [6]. RBCs are indeed constantly exposed to large amounts of ROS produced from different intracellular and extracellular sources in the vasculature, which can interfere with HbNO complex accumulation. Among these various sources, ROS can be generated by autoxidation of ferrous ion in partially oxygenated hemoglobin to ferric hemoglobin (metHb) with the release of O_2_^.-^ [10]. The subsequent oxidation of metHb by H_2_O_2_ or another strong oxidant such as peroxynitrite (itself formed by the direct reaction between NO and O_2_^.-^), can produce heme in the ferryl form (Hb -Fe^4+^) or globin radicals that potentiate pro-oxidant activity in RBCs (reviewed [23]). Beside the well-known role of Hb oxidation as ROS source [24], NADPH oxidases (NOXs) could also contribute to oxidative stress. Expression and activity of different isoforms of NOXs have largely been described in endothelial cells, smooth muscle cell, fibroblasts and immune cells [25] but little is known about NOXs in healthy erythrocytes. Recently, activity and catalytic subunits of NOX1 and NOX2 were identified in RBCs of patients with sickle cell disease, [11]. In our study, we identified immunoblotted signals for the catalytic subunits of NOX1, 2 and 5 isoforms in the erythrocyte membrane and cytoplasmic expression of NOX4 in RBCs from healthy volunteers (Fig. 1A). We excluded leukocyte contamination in our erythrocytes preparations by FACS analysis. We used VAS2870 to inhibit NOX2 and GKT137831, described as potent dual inhibitor for NOX1/NOX4 and recently for NOX5 [26,27]. With the caveat regarding specificity for these small molecule inhibitors, we observed that erythrocyte NOX1 and 2 produced most of the O_2_^.-^, detected with the CPH spin probe, in erythrocyte “ghost” membranes. Moreover, inhibition of NOX1 and NOX2 preserved the accumulation of HbNO in intact erythrocytes. Recently, the Ca^2+^ regulated NADPH oxidase, NOX5, was found to be overexpressed in RBCs during vascular diseases [28], as well as in endothelium and vascular smooth muscle cells during atherosclerosis, vascular inflammationn, angiogenesis [25,29] and in human cancers [30]. NOX4 is the only isoform that has been shown to produce H_2_O_2_ [31] and can have different physiological roles depending on stimulus and cell types [25]. These isoforms may contribute additional ROS production in erythrocytes under pathophysiological conditions.

In the membrane fraction, O_2_^.-^ could directly react with NO (produced either from paracrine sources or eNOS expressed in the erythrocyte membrane, as shown by us and others [7,32,33], to form the potent oxidant, peroxynitrite (ONOO-). ONOO-, in turn, can produce partial eNOS uncoupling (e.g. by oxidizing its cofactor BH_4_; or by eNOS S-glutathionylation [[34], [35], [36]]), which we detected from the inhibitory effect of l-NAME (Fig. 1B), or directly oxidize and degrade HbNO. However, it is important to emphasize that NOX produce O_2_^.-^ in a polarized way, i.e. across the membrane, towards the extracellular space in the present case. There, O_2_^.-^ is rapidly dismutated to form H_2_O_2_ by the extracellular SOD3, which is clearly detected around the intact erythrocyte membrane (Fig. 2B). Accordingly, suppression of O_2_^.-^ production (by inhibition of NOX1-2 and uncoupled eNOS), or its dismutation (by the constitutively present or exogenously added SOD3; Fig. 1B and C) protect HbNO from degradation.

However, H_2_O_2_ itself is an oxidant and, according to conventional thinking, can migrate across cell membranes and, in the case of RBCs, could also react with hemoglobin, as well as other protein cysteine residues with low pKa [37], and interfere with HbNO accumulation. By oxidizing the ferrous and ferric forms of hemoglobin, H_2_O_2_ can also produce the highly reactive Fe(IV) ferrylhemoglobin, however such formation was shown to produce only small changes in the structure and property of the membrane of isolated erythrocytes [38], owing probably to high erythrocyte antioxidant activity. The converse, i.e. increased HbNO from activation of eNOS by H_2_O_2_, as previously shown in endothelium [[39], [40], [41]], is not supported from our data, at least in erythrocytes. What, then, of the effect of extracellular H_2_O_2_ on erythrocyte redox balance and HbNO?

Considering the numerous different sources of oxidant to which they are exposed, it is not surprising that erythrocytes are equipped with an extensive intracellular antioxidant defense system [14,23,42]. H_2_O_2_, also produced from the dismutation of O_2_^.-^ by the cytosolic SOD1, can be decomposed by catalase, glutathione peroxidase and by different isoforms of peroxiredoxin (Prdx1, 2 and 6). We found immunoblotted signals for all these enzymes in the cytosolic lysates of human RBCs (Fig. 2A). Prdx1 and 6 were similarly identified in RBCs, at lower abundance compared to Prdx2, the most abundant of these isoforms [43]. Accordingly, Prdx2 knockout mice developed hemolytic anemia and Heinz body formation already at 5 weeks, while Prdx1 knockout mice showed the onset of malignant tumor and Heinz body hemolytic anemia only after 9 months of age; no erythrocyte abnormalities were reported in mice genetically deleted for Prdx6 [19,44]. On the other hand, catalase was described as the major enzyme responsible for the degradation of exogenous H_2_O_2_ in erythrocytes [45,46]. Indeed, RBCs from catalase knockout mice were exquisitely sensitive to exogenously applied H_2_O_2_, that induced metHb formation, contrary to RBCs from Prdx2 knockout mice, although the latter displayed constitutively (i.e. in absence of added H_2_O_2_) increased intracellular H_2_O_2_, suggesting that Prdx2 mainly controls intracellular H_2_O_2_ formation [19,47]. Our own data would be in line with this paradigm, as inhibition of Prdx2 (by ConoidinA), but not catalase (with 3-AT) increased the constitutive (i.e. without exogenous H_2_O_2_) ROS detection (Fig. 3A); whereas, upon addition of H_2_O_2_, ROS accumulation rose more rapidly in cells after inhibition of catalase compared with Prdx2 inhibition (Fig. 3B). The slower kinetics are probably due to the slow reduction cycle of dimerized Prdx2 by a thioredoxin reductase [48]. Over time (45 min), inhibition of each enzyme results in the same level of intracellular ROS, suggesting a participation, albeit delayed, of the (mostly cytosolic) Prdx2 (Fig. 3B). In fact, others showed that Prdx2 contributed to the reduction of peroxynitrite [49], and was even able to associate to the plasma membrane where it reduced lipid hydroperoxides and neutralized ROS produced by Hb autoxidation [50]. This probably explains the observed increase in heme degradation in Prdx2 knockout mice [16] and the decay in HbNO stability under constitutive ROS production after ConoidinA treatment (Fig. 3C) in our hands. The inhibition of catalase by 3-AT similarly decreased the HbNO steady-state levels under basal conditions (Fig. 3C), but had a more consistent effect than Prdx2 inhibition after addition of exogenous H_2_O_2_ up to 15min (Fig. 3D), consistent with the early difference in kinetics of ROS accumulation in Fig. 3B. The other enzymatic systems probably relay these first two antioxidants at later time points. Indeed, GPx1 that we also found to be expressed in cytosolic fractions of RBCs (Fig. 2A), was shown to participate in the reduction of exogenous H_2_O_2_ [47]. Consistent with our observations upon inhibition of endogenous catalase, we found that pre-incubation with extracellular (non-PEGylated, i.e. membrane impermeable) catalase decreased intracellular ROS and preserved HbNO formation and stability in human erythrocytes incubated with exogenous H_2_O_2_ (Fig. 4). Of note, this was reproduced with extracellular SOD or catalase in erythrocytes at 21% of O_2_, demonstrating the preservation of HbNO by these enzymes under constitutive production of ROS at air oxygen level (Fig. 4D). By reducing O_2_^.−^extracellularly, SOD was expected to preserve HbNO accumulation but so did catalase, suggesting that constitutively produced H_2_O_2_ is a significant extracellular oxidant species limiting intracellular HbNO formation. Contrary to conventional thinking, the transmembrane passage of H_2_O_2_ may not be entirely passive. Specific members of the family of membrane proteins, Aquaporins, were recently proposed as candidates for the facilitated transport of exogenous H_2_O_2_ across plasma membranes [51]. As one of the most abundant proteins expressed in erythrocytes, Aquaporin1 would likely be involved in this process. Indeed, molecular modeling of Aquaporin1 suggested that the central water pore may allow the passage of H_2_O_2_ in addition to water, and recent evidence demonstrated that murine aquaporin 1 facilitated H_2_O_2_ migration into different cells [[51], [52], [53], [54]].

Our model has some limitations, i.e. from our use of a relatively high (50  μM) concentration of NONOate which may seem to deviate from (patho)physiological concentrations of endogenously produced NO. Note, however, that the final concentration of bioavailable NO in solution may differ from that calculated from the NO donor (in fact, using spin-trapping EPR spectroscopy, we found it not to exceed 20% of the calculated dose in our experimental conditions). Nevertheless, this concentration allowed us to efficiently test the dynamic formation of HbNO in intact erythrocytes with the optimal signal/noise ratio. It is also important to note that we observed reproducible HbNO signals in the range of 100–200 nmol/l in intact erythrocytes freshly drawn from normal volunteers, in absence of incubation with exogenous NO donors [4]. While more prospective studies in large cohorts of patients will be needed to establish the validity of HbNO measurement as a biomarker of vascular health, our study identifies critical redox mechanisms influencing the dynamic formation of HbNO which are likely to be even more prevailing in erythrocytes from patients with cardiovascular risk factors.

In conclusion, our work shows that the steady-state level of HbNO is profoundly influenced by the redox balance between pro-oxidant and anti-oxidant enzymatic systems in human erythrocytes. In particular, we identified a significant contribution of extracellular H_2_O_2_, the transport of which may be facilitated by Aquaporin-1 richly expressed in the erythrocyte membrane. This points to HbNO measurements as indicators of vascular oxidant stress, as well as NO bioavailability and potentially, as useful biomarkers of early endothelial dysfunction.
