# Supplementary material for: Redox regulation of nitrosyl-hemoglobin in human erythrocytes
Source: Redox Biol. 2019 Dec 5;34:101399. doi: 10.1016/j.redox.2019.101399 (PMC7327715; doi:10.1016/j.redox.2019.101399)
Supplement: Multimedia component 1 [file mmc1.docx]

**Supporting figures**

**
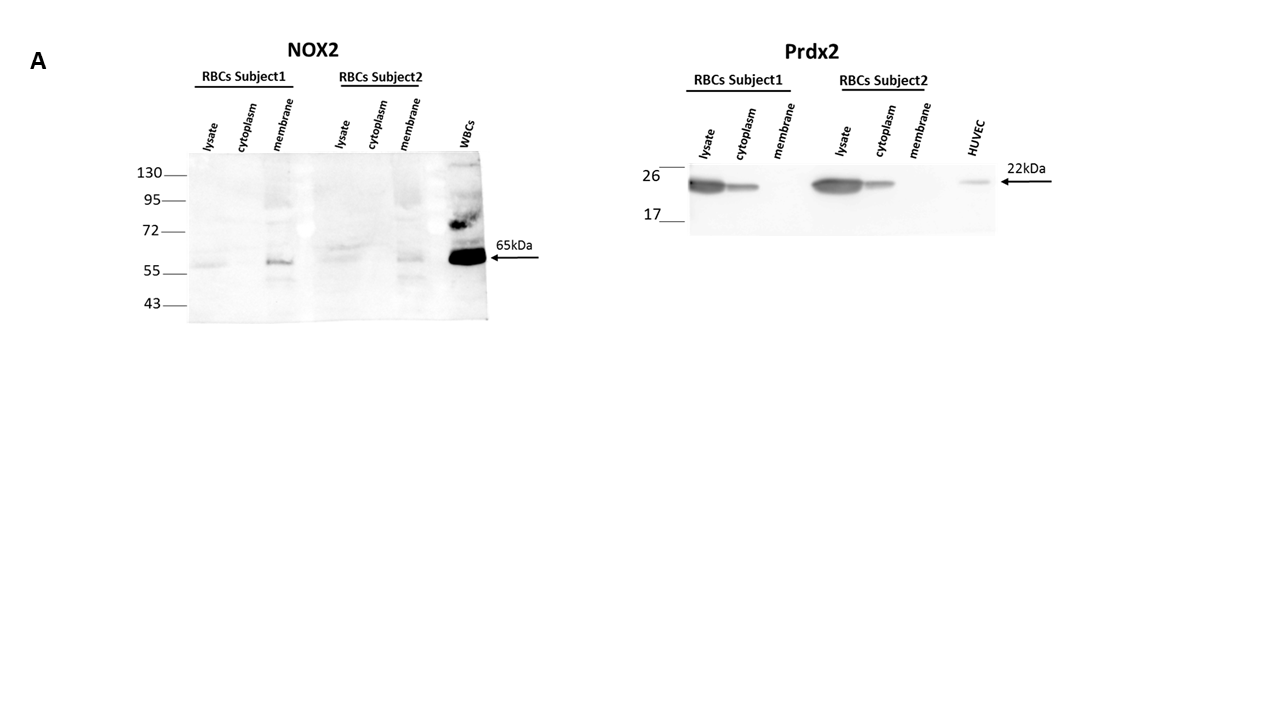
**


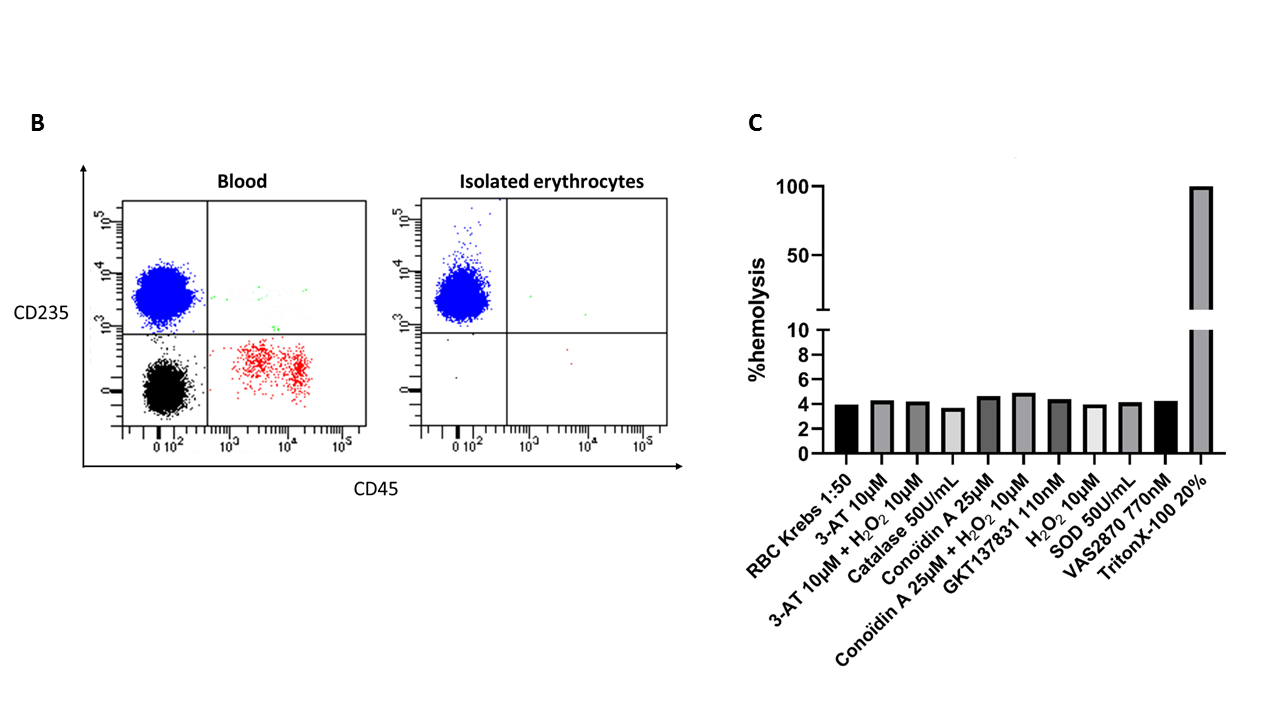


S1 Figure.

A) Representative western blots detected with chemiluminescent substrate for alkaline phosphatase. RBCs lysates, RBC cytosolic fractions, and isolated ghost membranes (50 and 20 µg of protein each respectively for NOX2 and Prdx2) reveal the expression of NOX2 at ~65kDa in membrane fractions and of Prdx2 at ~22kDa in cytosolic fractions of human RBCs from healthy subjects. Human WBCs lysate was loaded as a positive control for NOX2 and HUVEC cell lysate for Prdx2.

B) Representative flow cytometric two parameter dot plot of blood and isolated RBCs from a healthy subject co-stained with anti-CD235a (Glycophorin-A)-FITC and anti-CD45-APC antibodies. The left panel shows blood staining of erythrocytes (in blue; CD235 positive) and leukocytes (in red; CD45 positive). The right panel shows the exclusive identification of RBCs as CD235-positive events in the upper left quadrant excluding leukocytes contamination in erythrocytes isolated as described in Materials and Methods.

C) Hemolysis assay of isolated RBCs incubated with different drugs: antioxidant inhibitors (3-AT or Conoidin A) with/without H_2_O_2_, NOX inhibitors (GKT137831 or VAS2870), H_2_O_2_ only, and antioxidants (SOD or catalase). RBCs treated with 20% of TritonX-100 are used as a positive control. n = 3 different RBC preparations.


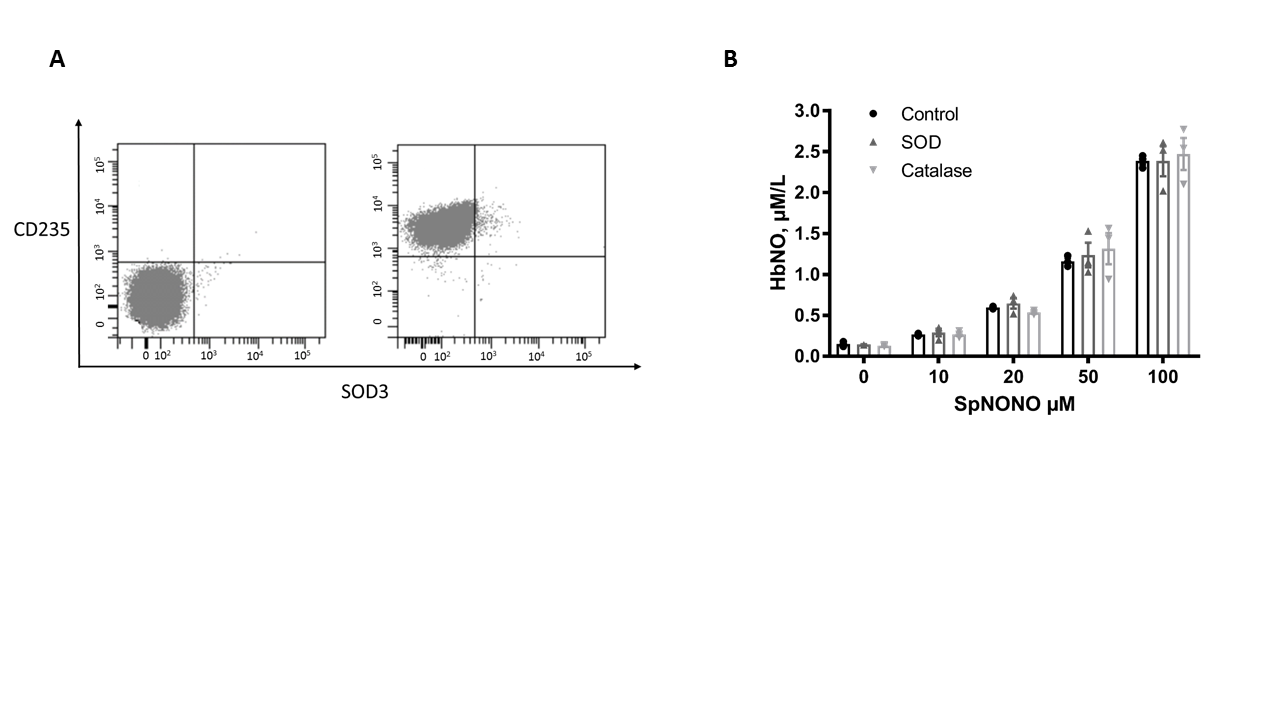


S2 Figure.

A) Representative flow cytometric two parameter dot plot of isolated RBCs from a healthy subject co-stained with primary anti-CD235a (Glycophorin-A) and secondary Alexa Fluor-488-conjugated anti-IgG antibodies; and primary anti-SOD3 and secondary Alexa Fluor-647-conjugated anti-IgG antibodies. The left panel shows co-staining only with conjugated secondary antibodies and the right panel shows the identification of RBCs as SOD3–positive and CD235a-positive events in the upper right quadrant.

B) HbNO concentrations in human RBCs reconstituted at 50% of haematocrit and incubated with SOD or catalase or vehicle at 1% of O_2_ for 1 hour, followed by 45 minutes incubation with graded concentrations of Spermine-NONOate before freezing for the low-temperature EPR spectroscopy.

Data are shown as mean values ± SEM and treated using a mixed model statistical analysis with Dunnett’s adjustment for multiple comparisons; n =3 different RBC preparations.
